# Invasion of ovarian cancer cells is induced byPITX2-mediated activation of TGF-β and Activin-A

**DOI:** 10.1186/s12943-015-0433-y

**Published:** 2015-08-23

**Authors:** Moitri Basu, Rahul Bhattacharya, Upasana Ray, Satinath Mukhopadhyay, Uttara Chatterjee, Sib Sankar Roy

**Affiliations:** Cell Biology and Physiology Division, CSIR-Indian Institute of Chemical Biology, Council of Scientific and Industrial Research, 4 Raja S. C. Mullick Road, Kolkata, 700032 India; Department of Endocrinology and Metabolism, IPGMER and SSKM Hospital, 244 AJC Bose Road, Kolkata, India; Department of Pathology, IPGMER and SSKM Hospital, 244 AJC Bose Road, Kolkata, India

**Keywords:** PITX2, TGF-β signalling, Activin-A, Invasion, EMT, IOSE, Ovarian cancer cells

## Abstract

**Background:**

Most ovarian cancers are highly invasive in nature and the high burden of metastatic disease make them a leading cause of mortality among all gynaecological malignancies. The homeodomain transcription factor, PITX2 is associated with cancer in different tissues. Our previous studies demonstrated increased PITX2 expression in human ovarian tumours. Growing evidence linking activation of TGF-β pathway by homeodomain proteins prompted us to look for the possible involvement of this signalling pathway in PITX2-mediated progression of ovarian cancer.

**Methods:**

The status of TGF-β signalling in human ovarian tissues was assessed by immunohistochemistry. The expression level of *TGFB/INHBA* and other invasion-associated genes was measured by quantitative-PCR (Q-PCR) and Western Blot after transfection/treatments with clones/reagents in normal/cancer cells. The physiological effect of PITX2 on invasion/motility was checked by matrigel invasion and wound healing assay. The PITX2- and activin-induced epithelial-mesenchymal transition (EMT) was evaluated by Q-PCR of respective markers and confocal/phase-contrast imaging of cells.

**Results:**

Human ovarian tumours showed enhanced TGF-β signalling. Our study uncovers the PITX2-induced expression of *TGFB1*/*2*/*3* as well as *INHBA* genes (*p* < 0.01) followed by SMAD2/3-dependent TGF-β signalling pathway. PITX2-induced TGF-β pathway regulated the expression of invasion-associated genes, *SNAI1*, *CDH1* and *MMP9* (*p* < 0.01) that accounted for enhanced motility/invasion of ovarian cancers. Snail and MMP9 acted as important mediators of PITX2-induced invasiveness of ovarian cancer cells. PITX2 over-expression resulted in loss of epithelial markers (*p* < 0.01) and gain of mesenchymal markers (*p* < 0.01) that contributed significantly to ovarian oncogenesis. PITX2-induced *INHBA* expression (*p* < 0.01) contributed to EMT in both normal and ovarian cancer cells.

**Conclusions:**

Overall, our findings suggest a significant contributory role of PITX2 in promoting invasive behaviour of ovarian cancer cells through up-regulation of *TGFB*/*INHBA*. We have also identified the previously unknown involvement of activin-A in promoting EMT. Our work provides novel mechanistic insights into the invasive behavior of ovarian cancer cells. The extension of this study have the potential for therapeutic applications in future.

**Electronic supplementary material:**

The online version of this article (doi:10.1186/s12943-015-0433-y) contains supplementary material, which is available to authorized users.

## Background

Ovarian cancer is a highly metastatic disease and is the leading cause of death among all gynaecological malignancies [1]. Unfortunately, the disease is often detected at an advanced stage (stages III-IV) and progresses very rapidly, as it acquires an aggressive phenotype. A number of growth factors, including transforming growth factor-β (TGF-β) regulate the proliferation of ovarian surface epithelial (OSE) cells and increases metastasis [2]. TGF-β binds to its specific receptors, type-I (TβRI) and type-II (TβRII). Upon ligand binding, TβRII trans-phosphorylates and activates TβRI, which in turn phosphorylates Smad2 and Smad3 [3, 4]. The latter two then bind to Smad4 and the complex translocates to the nucleus to regulate the expression of target genes. Other member of the TGF-β superfamily, like activin transduces signals mainly through the SMAD2/3-dependent pathway [5]. *INHBA* forms a disulfide-linked homodimer, known as activin-A which is a polypeptide hormone of primarily gonadal origin [6, 7]. The mojor gonadal sites of its production is Sertoli cells of males and ovarian granulosa cells of female origin [6, 7]. High levels of activin-β_A_ subunit is detected in majority of the patients with granulosa cell tumors [8], but almost absent in ovarian epithelial tumors except mucinous carcinoma [9]. In addition, increased expression of activin-A is observed in esophageal [10] and colorectal carcinomas [11]. High expression of activin-A was found in stage IV colorectal cancer [12] and correlated with poor overall survival rate [11, 12]. However, there are no reports on the regulation of activin-A and its role in epithelial ovarian cancer progression.

Highly invasive and metastatic behavior underpin the aggressive nature of ovarian cancers. Epithelial-mesenchymal transition (EMT) is a major mechanism for the conversion of early-stage tumors to invasive malignancies due to the loss of epithelial adherence and tight junctions [13, 14]. Transcription factor like Snail acts as a key regulator in the induction of cellular invasion, in part, by suppressing the expression of the epithelial specific adhesion molecule, E-cadherin and by increasing the expression of matrix metalloproteinases MMPs; [15]. TGF-β-signalling, on the other hand, enhances the invasive properties of ovarian cancers partially through up-regulation of MMPs [16].

The homeobox genes are widely implicated in various human cancers, acting as oncogenes or tumour suppressors [17–21]. Pituitary homeobox 2 (*PITX2*), a member of the bicoid/paired-like homeobox gene family, is a multifunctional transcription factor [22–24]. Three different isoforms of PITX2 (PITX2A/B/C), differ only in their amino terminus and regulate the transcription of target genes differentially [25]. Recently, several reports highlighted the association of *PITX2* with progression of breast and colorectal cancers [26, 27]. We observed the up-regulated expression of PITX2 in ovarian tumours [28] and simultaneously we found induced TGF-β signaling pathway in the same tissue sections. Considering the importance of TGF-β signalling pathway in promoting oncogenesis of several tissues, we aimed to investigate possible involvement of PITX2 in promoting invasiveness of ovarian cancer cells through the regulation of TGF-β signalling pathways. We also explored the role of activin-A in the progression of epithelial ovarian cancers.

## Results

### Activation of TGF-β signalling pathway in human ovarian tumours

**Fig. 1:**
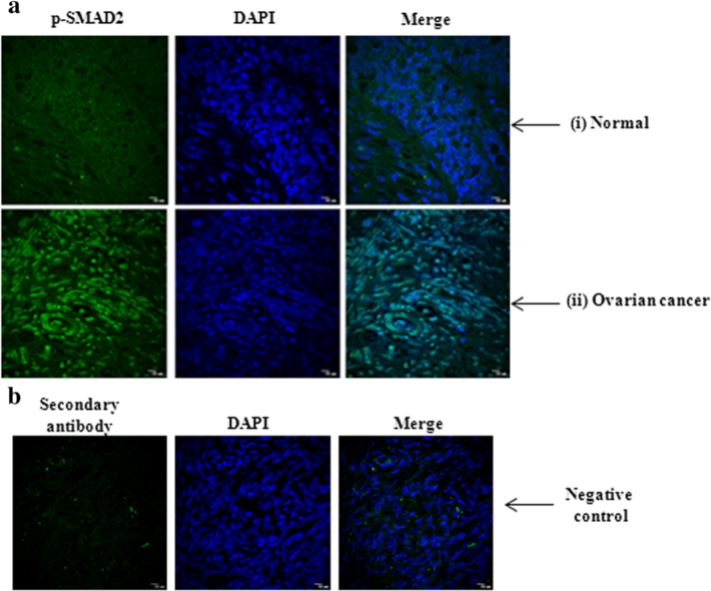
The expression of p-SMAD2 is up-regulated in human ovarian cancer. **a** The level of p-SMAD2 was shown by IHC in human ovarian tissue-sections with p-SMAD2 antibody followed by Alexa Fluor-488 (green) of normal (i; *n* = 20) and ovarian tumor (ii; *n* = 20), out of which 15 samples are of high grade and 5 samples are benign. The DAPI-stained nuclei and the merged images were also shown. **b** The negative control images represent the staining in presence of secondary antibody and DAPI but without primary antibody. The images were taken at the same exposure time. Scale bar, 10 μm

We evaluated the status of TGF-β signalling in ovarian tumours. The level of phospho-SMAD2, (as readout of active TGF signalling; Fig. 1a) was measured by immunohistochemical analysis. Confocal imaging of tissue sections showed intense staining of p-SMAD2 in human ovarian cancer (ii) compared to normal (i) tissues, supporting the activation of TGF-β signalling pathway. The specificity of the staining was checked by staining the sections in presence of secondary antibody only and DAPI without primary antibody (Fig. 1b). Simultaneously, the increased expression of PITX2 was observed in the same ovarian tumor sections (i) compared to normal (ii) (Additional file 1: Figure S1).

### SMAD-dependent TGF-β signalling is activated by PITX2 in ovarian cancer cells

**Fig. 2:**
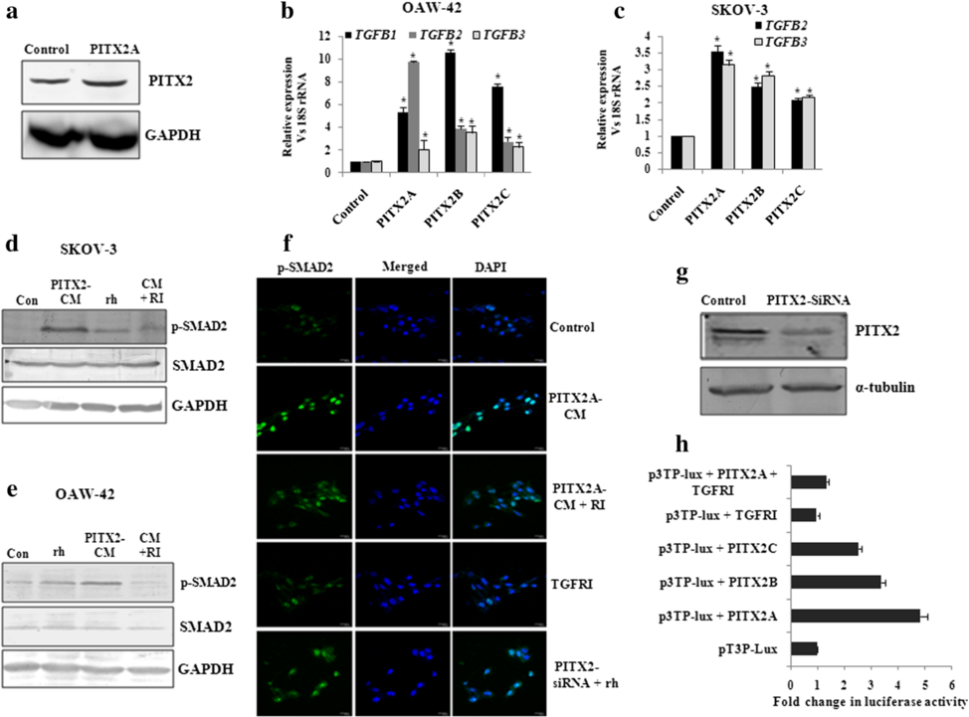
PITX2 induces TGF-β signalling pathway in ovarian cancer cells. **a** Western blot was performed with the lysate of OAW-42 cells transiently transfected with *PITX2A* expression clone. **b-c** Q-PCR assay of *TGF-B1/B2/B3* (for OAW-42 cells; **b**) and *TGF-B2/B3* (for SKOV-3 cells; **c**) was done with specific primers with RNA isolated from PITX2-overexpressed respective cells. The comparative expression of respective genes is shown as relative ‘fold’ change (mean ± S.E.M). * represents *p* < 0.05. **d-e** The conditioned-medium (PITX2-CM) was collected after transient transfection with *PITX2A*. Freshly plated serum-starved SKOV-3 (**d**) and OAW-42 (**e**) cells were incubated for 2 h with PITX2-CM alone or in combination with 20 ng/ml TGFRI (RI) followed by Western blot of the lysates with p-SMAD2 and SMAD2 antibodies. The lysate of the cellstreated with rhTGF-β1 (rh; for 30 min) was blotted with the respective antibodies. Here, GAPDH was used as loading control. **f** Confocal imaging for p-SMAD2 was performed in SKOV-3 cells treated or transfected as mentioned, where the left panel shows the p-SMAD2 expression, DAPI-stained nuclei in the middle panel and the right panel shows their merged image. The images were taken at the same exposure time. Scale bar, 20 μm. **g** Lysates were prepared from PITX2A-siRNA trasfected OAW-42 cells for Western blot with PITX2-antibody. Here, α-tubulin was used as loading control. **h** OAW-42 cells were transfected with p3TP-Lux vector alone or along with expression constructs of *PITX2* isoforms or pcDNA3 (empty vector) and treated with TGFRI for 16 h for luciferase assay. The activities are shown as mean fold enhancement compared to the p3TP*-*construct without *PITX2* expression after normalization with renilla luciferase activity

Considering the association of homeodomain proteins in activating TGF-β signalling in different cancer types, we attempted to investigate whether PITX2 could activate the same in ovarian cancer cells. For that, the increased expression of PITX2 was verified by Western blot with lysate of PITX2A-transfected OAW-42 cells (Fig. 2a). The ectopic over-expression of *PITX2A/B/C* significantly enhanced the mRNA levels of *TGF-B1*/*B2*/*B3* in OAW-42 (*p* < 0.01; Fig. 2b) cells as shown by Q-PCR assay. Similarly, increase in *TGF-B2/B3* was observed in SKOV-3 (*p* < 0.01; Fig. 2c) cells upon over-expression of PITX2 isoforms. The incubation of freshly cultured cells with the conditioned medium of *PITX2A*-transfected cells (PITX2-CM) induced the p-SMAD2 level. This induction was reduced in presence of TGF-β receptor inhibitor (TGFRI), suggesting the involvement of PITX2-mediated activation of TGF-β signalling pathway both in SKOV-3 (Fig. 2d) and OAW-42 cells (Fig. 2e). Here, the treatment of cells with rhTGFβ-1 also activated the p-SMAD2 level as a mark of positive control (Fig. 2d-e). The nuclear p-SMAD2 level was enhanced by the treatment of SKOV-3 cells with PITX2-CM as observed by confocal imaging using specific antibody (Fig. 2f). In contrast, treatment with rhTGFβ1 in PITX2-siRNA transfected cells reduced the intensity of expression. Here, the reduction in PITX2 protein was confirmed by Western blot with PITX2-siRNA trasfected cell lysate (Fig. 2g). Further, the ectopic over-expression of *PITX2A/B/C* augmented the activity of TGFβ/SMAD-responsive reporter construct (p3TP-lux) by 3-5 folds (Fig. 2h) in OAW-42 cells, however, TGFRI-treatment suppressed this trans-activation (Fig. 2h). Taken together, the results suggest activation of TGF-β signalling pathway by PITX2.

### PITX2 contributes to the EMT and invasion of ovarian cancer cells

**Fig. 3:**
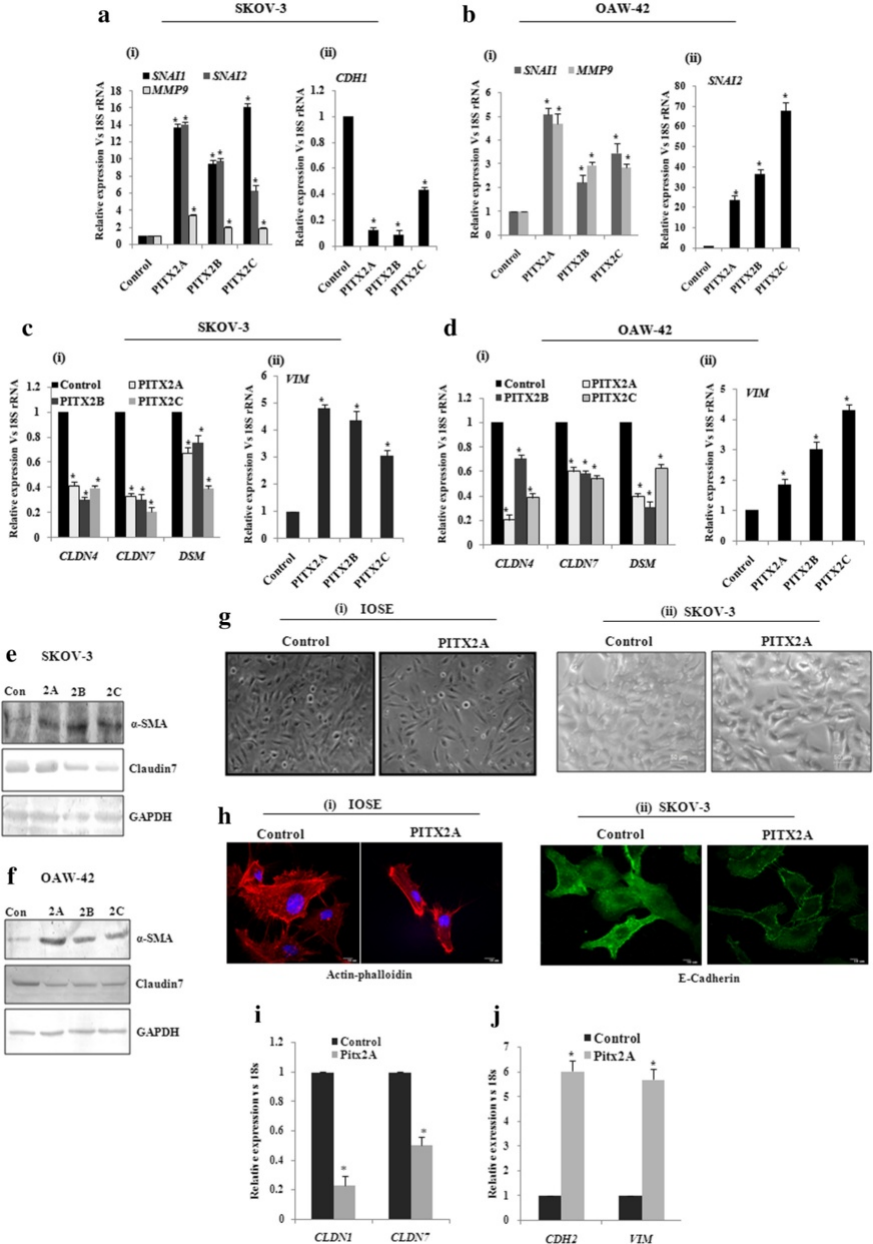
Over-expression of PITX2 affects markers for EMT and invasion in ovarian cancer cells. **a-d** The expression level of *SNAI1, SNAI2, MMP9*, *CLD4, CLD7, DSM* and *VIM* genes were quantified by Q-PCR assay after ectopic over-expression of *PITXA/B/C* into SKOV-3 (**a** and **c**) and OAW-42 (**b** and **d**) cells. The expression of *CDH1* was assessed in SKOV-3 cells (Fig A-ii) upon transient transfection of PITX2 isoforms. The comparative expression of respective genes is shown as relative ‘fold’ change (mean ± S.E.M). **e-f** Western blot analysis of the proteins with respective antibodies was performed with the lysates of *PITX2*-tranfected SKOV-3 (**e**) and OAW-42 (**f**) cells. Here, GAPDH was used as internal loading control. **g** Bright field microscopy images exhibit the epithelial phenotype of the control and mesenchymal phenotype of PITX2A-transfected IOSE (G-i) and SKOV3 (G-ii) cells cells. **h** Phalloidin staining of IOSE cells (**i**) and imunofluorescence imaging of E-cadherin was performed for the SKOV-3 cells (ii). **i-j** Q-PCR assay was performed to check the expression of *CLDN1*, *CLDN7*, *CDH2* and *VIM* in PITX2-overexpressed IOSE cells. *represents *p* < 0.05

In conjunction with our earlier finding of the up-regulated expression of PITX2 in human ovarian tumors [20], we attempted to check the regulation of invasion-associated genes by PITX2. The transient transfection of *PITX2* isoforms significantly increased the expression of these genes like Snail (*SNAI1*), Slug (*SNAI2*) and *MMP9* in both SKOV-3 (Fig. 3a) and OAW-42 cells (Fig. 3b). In contrast, PITX2 over-expression reduced the mRNA level of E-cadherin (Fig. 3a-ii) in OAW-42 cells. Since EMT is an essential prerequisite for the metastasis of ovarian cancer, we tried to elucidate the role of PITX2 over-expression in inducing EMT. PITX2 isoforms significantly reduced (*p* < 0.01; Fig. 2c-i) the expression of epithelial marker genes claudin-4 (*CLD4*), claudin-7 (*CLD7*) and desmoplakin (*DSM*), whereas the mesenchymal gene, vimentin (*VIM*; Fig. 3c-ii) was induced by all isoforms of *PITX2* in SKOV-3 cells. Similarly, the expression of *CLD4*, *CLD7* and *DSM* (Fig. 3d-i; *p* < 0.05) were reduced while that of *VIM* (Fig. 3d-ii; *p* < 0.005) was up-regulated in PITX2-overexpressed OAW-42 cells. Consistently, the expression of α-smooth muscle actin (α-SMA, a mesenchymal marker) was more intense in PITX2-transfected SKOV-3 (Fig. 3e) and OAW-42 (Fig. 3f) cells as shown by Western-immunobloting. However, the protein level of claudin-7 was remarkably reduced in PITX2 over-expressed SKOV-3 (Fig. 3e) and OAW-42 (Fig. 3f) cells. The EMT-like phenotypical changes in IOSE cells were verified by bright field microscopy (Fig. 3g-i) and the actin rearrangement was observed by phalloidin staining (Fig. 3h-i). SKOV-3 cells also showed phenotypic changes upon PITX2A-overexpression in phase contrast microscopy (Fig. 3g-ii). A significant decrease in E-cadherin was observed by immunostaining in PITX2A-transfected cells compared to controls (Fig. 3h-ii). There was a significant decrease (*p* < 0.01; Fig. 3i) of the epithelial markers *CLDN1* and *CLDN7,* while mesenchymal markers like N-cadherin (*CDH2*) and *VIM* showed ~ 4.5-5.5 fold up-regulation (Fig. 3j) in IOSE cells transfected with PITX2A. Similar changes in parameters primarily observed during EMT were also noticed in PITX2 over-expressed cells.

### Migration and invasion of ovarian cancer cells are enhanced by PITX2 through TGF-β pathway

**Fig. 4:**
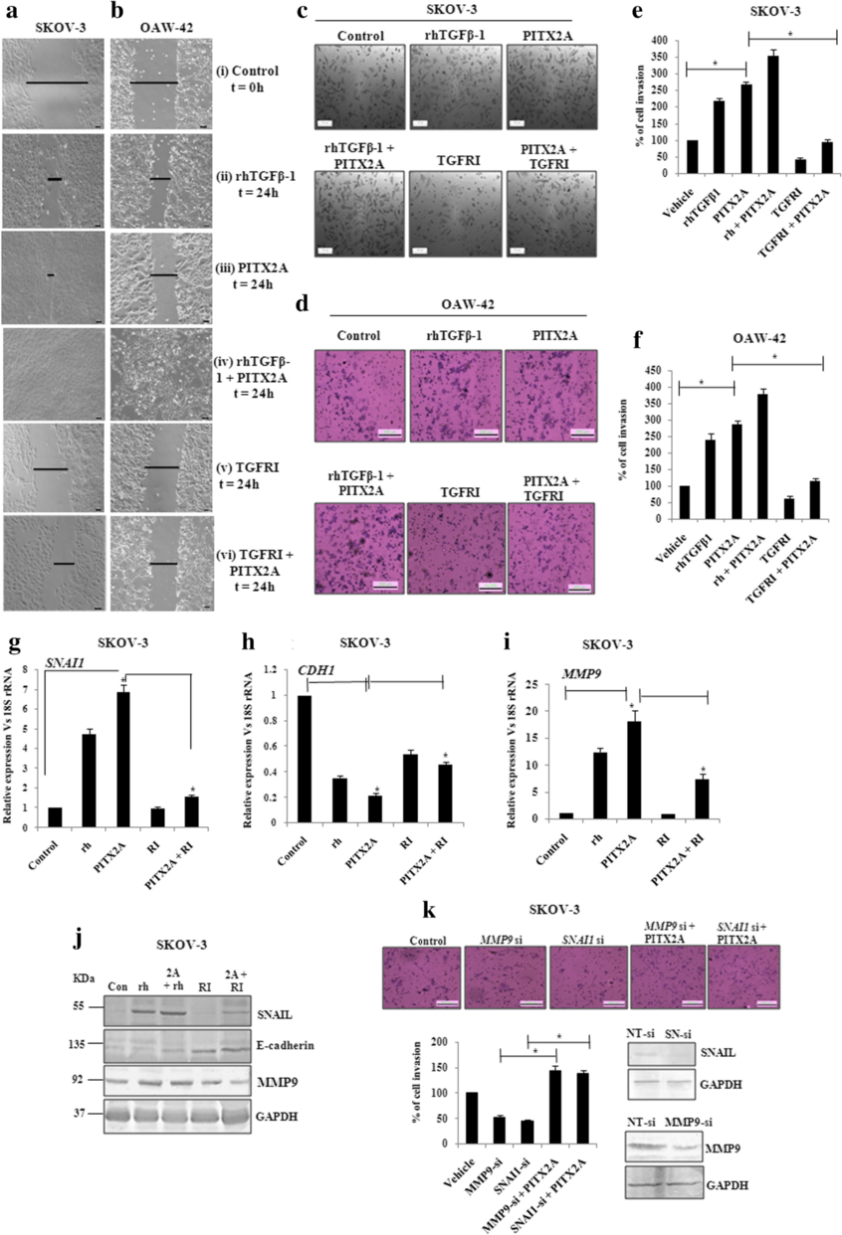
The regulation of invasion and migration is served by PITX2-mediated activation of TGF-β signalling pathway. Wound healing assay was performed of SKOV-3 (**a**) and OAW-42 (**b**) cells after treatment with rhTGFβ1 (ii), transient transfection of *PITX2A* (iii) and treatment with rhTGFβ1 (iv) or TGFRI (v) of PITX2-transfected cells in the time course of 24 h. T = 0 h at control cells (i) signifies the time of scratching the cells with pipette tips. The arrows indicate the width of wound and the assay was repeated three times independently. Scale bar: 50 μm. Transwell migration and invasion assay was performed in SKOV-3 (**c**) and OAW-42 (**d**) cells after treatment and/or transient transfection as mentioned. Scale bar: 200 μm. Cells at three independent fields for each well were counted and plotted with error bar for SKOV-3 (**e**) and OAW-42 (**f**) cells. **g-i** pcDNA3 or *PITX2A-*transfected SKOV-3 cells were treated with rhTGFβ1 (10 ng/ml) or TGFRI (20 ng/ml) followed by isolation of RNA and Q-PCR with the primers of *SNAI1* (**g**), *CDH1* (**h**) and *MMP9* (**i**). Relative gene expression is indicated as ‘fold’ change in the Y-axis (mean ± SEM). * represents *p* < 0.01. **j** Lysates of the cells transiently transfected and/or treated as indicated were immunoblotted with respective antibodies and the representative gel image was shown. **k** Transwell invasion assay was performed with SKOV-3 cells after transient transfection as mentioned (top). Cells at three independent fields for each well were counted and were plotted with error bar (bottom). The efficiency in knocking down the expression of SNAIL and MMP9 proteins by *SNAI*-(SN)-si and *MMP9*-si respectively was verified by Western blot of the transfected cell lysates with respective antibodies. Here, GAPDH was used as loading control (bottom)

To address the physiological significance of PITX2-mediated regulation of TGF-β pathway in promoting migration and invasion, wound healing assay was performed with SKOV-3 (Fig. 4a) and OAW-42 (Fig. 4b) cells. Treatment with rhTGF-β1 (ii) for 24 h or transient transfection of *PITX2A* (iii) for the same duration exhibited faster wound closure than the control set (i). Treatment of transiently transfected cells with rhTGF-β1 (iv) showed almost complete healing within the same period. Conversely, TGFRI-treatment (v) inhibited the healing, while ectopic over-expression of *PITX2A* slightly reduced its inhibitory effect (vi). The gap created by the wound before and after treatment/transfection has been depicted herewith. Matrigel invasion assay demonstrated ~2.5 folds increase (*p* < 0.005; Fig. 4c and e) in invasion of SKOV-3 cells upon transient transfection of *PITX2A;* the invasiveness was sharply reduced upon TGFRI treatment. In parallel, treatment of PITX2A-transfected cells with the rhTGF-β1 increased the invasion by ~3.5 folds (*p* < 0.01; Fig. 4c and e). The above-mentioned effects were observed on OAW-42 cells (Fig. 4d and f) as well.

Further, the involvement of TGF-β signalling pathway on PITX2-mediated invasion and migration was verified by monitoring the expression of relevant genes . The expression of the *SNAI1* was increased ~ 7 folds (*p* < 0.05) in *PITX2A*-overexpressed SKOV-3 cells, which was reduced to < 2 folds (*p* < 0.01; Fig. 4g) by TGFRI. The rhTGFβ-1 alone induced the mRNA of *SNAI1* by ~ 4 folds (*p* < 0.01; Fig. 4g). In contrast, PITX2A-suppressed expression of *CDH1* was rescued by TGFRI-treatment by > 60 % (*p* < 0.05; Fig. 4h). Similarly, the *MMP9* expression was shown to be induced by ~ 16 folds (*p* < 0.01; Fig. 4i) by PITX2A-transfection. However, the TGFRI severely reduced it to ~ 8 folds (*p* < 0.05; Fig. 4i). In agreement to Q-PCR result, the Western-blot with specific antibodies supported the regulation at the protein level by TGF-β signalling pathway as well (Fig. 4j). To determine whether Snail and MMP9 contribute to PITX2-stimulated invasion, SKOV-3 cells were transfected with their respective siRNAs alone or along with *PITX2A*-expression vector followed by matrigel invasion assay. PITX2A induced the invasion of cells by > 2.5 folds (Fig. 3c), which was reduced by ~ 60 % (*p* < 0.01; Fig. 4k) by either *SNAI1*-or *MMP9*-siRNA transfection into PITX2A-overexpressed cells.

### PITX2-mediated up-regulation of Activin-A leads to increased p-SMAD2 levels

**Fig. 5:**
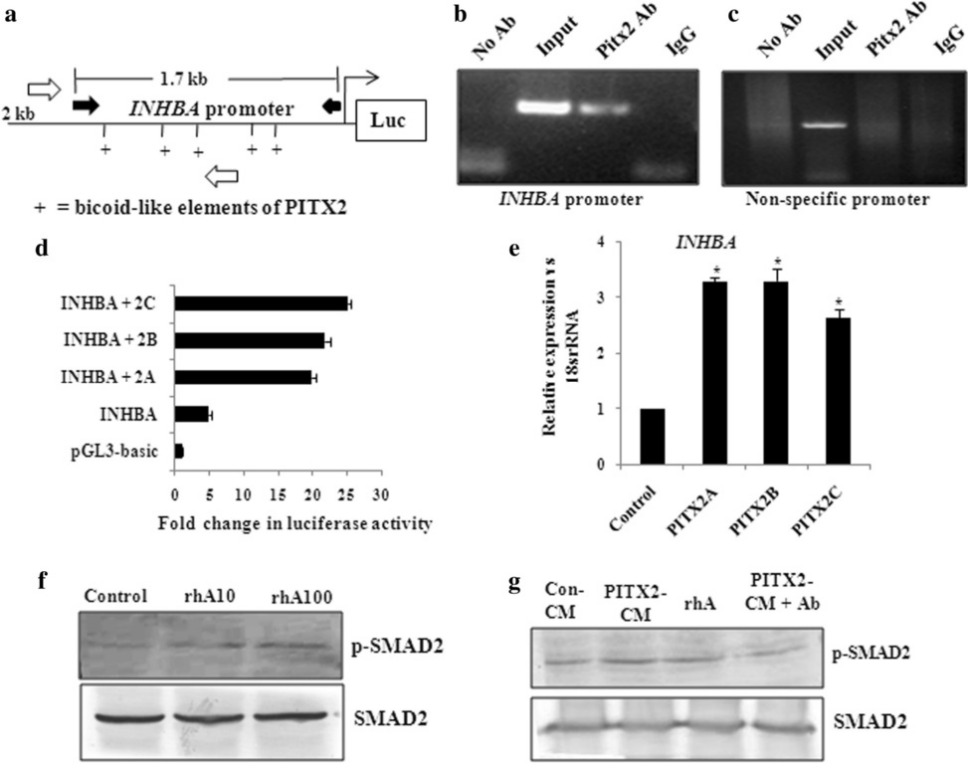
PITX2-regulated *INHBA* activates SMAD2 level. **a** Schematic diagram shows the 2 kb upstream region of *INHBA* promoter with the PITX2-specific bicoid-like (as +) elements. Here, the location of primers used to clone 1.7 kb region in pGL3 vector has been depicted with black solid arrow, while that used for ChIP-PCR has been shown with hollow arrows. **b-c** ChIP with SKOV-3 cells followed by the PCR showed the amplification of the *INHBA* promoter (**b**) from the chromatin input and from PITX2-IP DNA as indicated in the lanes. The PITX2 antibody used in this assay recognizes all three isoforms of PITX2. Amplification of respective promoters was not observed by PCR performed with IgG-IP and no antibody control sets. **c** Primers of an unrelated gene did not show amplification from PITX2-IP DNA. (**d**) The *INHBA* promoter as shown in Fig A was cloned into pGL3-basic vector. OAW-42 cells were transiently co-transfected with the construct alone or in combination with PITX2 isoforms followed by luciferase assay. The reporter activity of each clone was calculated in terms of fold change in PITX2-overexpressed cells compared with empty vector-transfected cells, after normalization with renilla luciferase activity. **e** Q-PCR analysis of the *INHBA* gene with specific primers was performed with the RNA isolated from *PITX2A/B/C*-transfected OAW-42 cells. Relative gene expression is indicated as ‘fold’ change in the Y-axis (mean ± SEM). The statistical analysis is done as described previously.* represents *p* < 0.05. **f** Western blot was performed to check the level of p-SMAD2 in OAW-42 cells after treatment with rhActivin-A (rhA10: 10 ng/ml and rhA100: 100 ng/ml) for 30 mins. **g** The levels of p-SMAD2 was assessed on treating the cells with PITX2A-conditioned medium (PITX2-CM), rhActivin-A (rhA: 100 ng/ml) or PITX2-CM in presence of activin neutralizing antibody (Pitx2-CM + Ab)

As p-SMAD2 level was higher in PITX2-CM treated cells than that of rhTGF-β1 (Fig. 2c), we checked for other PITX2-regulated ligands for possible explanation. Since several PITX2-specific cis-elements are present in the promoter of *INHBA* (Fig. 5a), we verified PITX2-mediated trans-activation of the same. ChIP assay with PITX2 antibody followed by PCR showed amplifications of the *INHBA* (Fig. 5b) promoter, indicating the binding of PITX2 with the respective promoter. The primers of an unrelated gene (*GAPDH* promoter) did not show any PCR amplification from the PITX2-IP DNA (Fig. 5c). All PCR products were sequenced to confirm their identities. Next, the promoter region of *INHBA* (Fig. 5a) was cloned into pGL3-basic vector. Transfection of OAW-42 cells with this reporter clone along with PITX2 expression constructs revealed activation of the promoter by ~20-25 folds (Fig. 5b) compared to transfection with the reporter construct alone. Q-PCR demonstrated significant up-regulation of the mRNA level of *INHBA* (Fig. 5e) upon ectopic over-expression of PITX2 isoforms. To find out the physiological effect of Activin-A, the homodimer form of INHBA, serum-starved OAW-42 cells were treated with recombinant protein. Westerm immunoblotting confirmed the induction of p-SMAD2 level in the cell lysates (Fig. 5f), indicating activation of TGF-β signalling pathway. Induction of p-SMAD2 was higher in cell lysates treated with 100 ng/ml of rhActivin-A compared to that treated with 10 ng/ml concentration. Therefore, the following experiments were done with 100 ng/ml rhActivin. Activin-A appeared to contribute directly to PITX2-induced p-SMAD2 level, as use of neutralizing antibody to Activin-A reduced the PITX2-CM-enhanced p-SMAD2 (Fig. 5g).

### Activin-A induces EMT and invasion in both normal and ovarian cancer cells

**Fig. 6:**
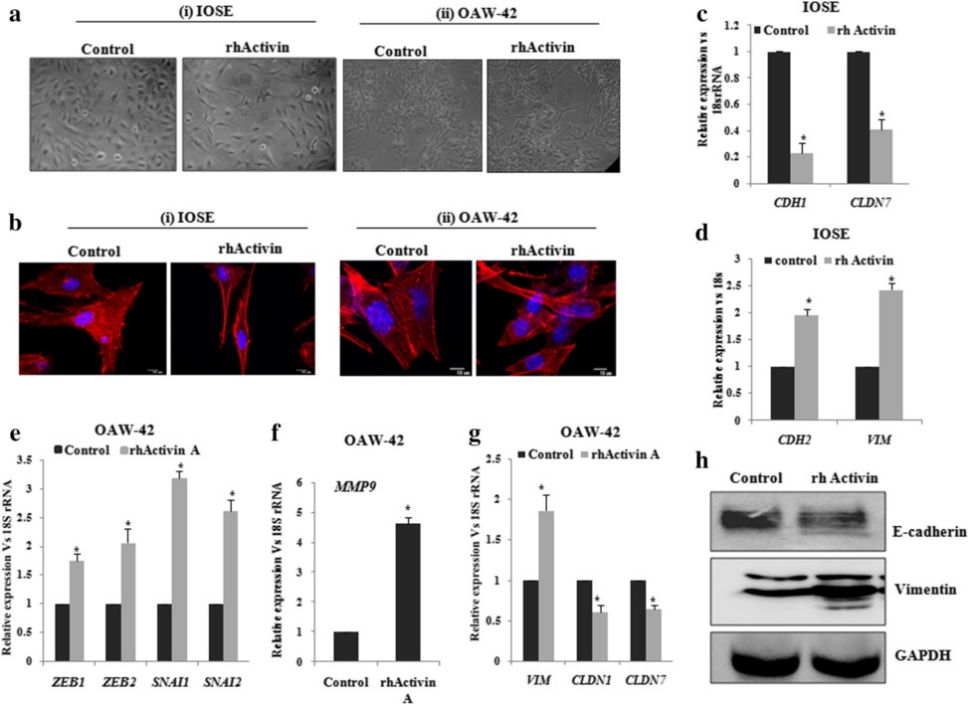
Activin-A promotes EMT in ovarian non-cancerous and cancer cells. **a-b** IOSE and OAW-42 cells were treated with rhActivin-A and their morphological changes were observed through bright field (A i-ii) as well as through confocal microscopy after phalloidin staining (B i-ii). **c-d** RNA was isolated from the IOSE cells to check the expression of *CDH1*, *CLDN7* (**c**) and *CDH2, VIM* (**d**) by Q-PCR. **e-g** RNA isolation was done from the OAW-42 cells to quantify *ZEB1*, *ZEB2,SNAI1*, *SNAI2* (**e**), *MMP9* (**f**), *CLDN1*,*CLDN7* and *VIM* (**g**) by Q-PCR with specific primers. Relative gene expression is indicated as ‘fold’ change in the Y-axis (mean ± SEM). The statistical analysis is done as described previously.*represents *p* < 0.05. **h** Western Blot was done with lysates of IOSE cells with respective antibodies as mentioned. GAPDH was used as the loading control

The trans-activation of *INHBA* gene by PITX2 (Fig. 5) made us curious whether it affects cellular invasiveness. Next, the treatment of IOSE and OAW-42 cells with rhActivin-A, the homodimer form of INHBA, resulted in the morphological changes (as checked by bright field microscopy; Fig. 6a i-ii) and actin rearrangements (through phalloidin staining followed by confocal microscopy; Fig. 6b i-ii) resembling that of mesenchymal cell type. For detailed study, we assessed the effect of activin-A on the expression of genes associated with cellular invasion and EMT. The expression of the EMT markers, including *CDH1*, *CLDN7* was decreased by ~ 60-70 % (Fig. 6c; *p* < 0.05) whereas the levels *CDH2* and *VIM* were increased by ~2 and 2.5 folds respectively (Fig. 6d) in IOSE cells. In addition, the expression of transcription factors including *SNAI1*, *SNAI2*, *ZEB1*, *ZEB2* was increased by ~ 2-3 folds (Fig. 6e, *p* < 0.01), while *MMP9* expression was found ~ 5 folds increase (Fig. 6f) by treatment of OAW-42 cells with rhActivin-A. In addition, reduction in *CLDN1* and *CLDN7* and increase in *VIM* was observed by activin-A treatment (Fig. 6g). The change in the protein levels of VIM and E-cadherin by rhActivin-A (Fig. 6h) was consistent with Q-PCR data of the same in IOSE. Thus, overall findings clearly indicate the involvement of activin in promoting EMT.

### Activin-A contributes to PITX2-induced cellular invasion

**Fig. 7:**
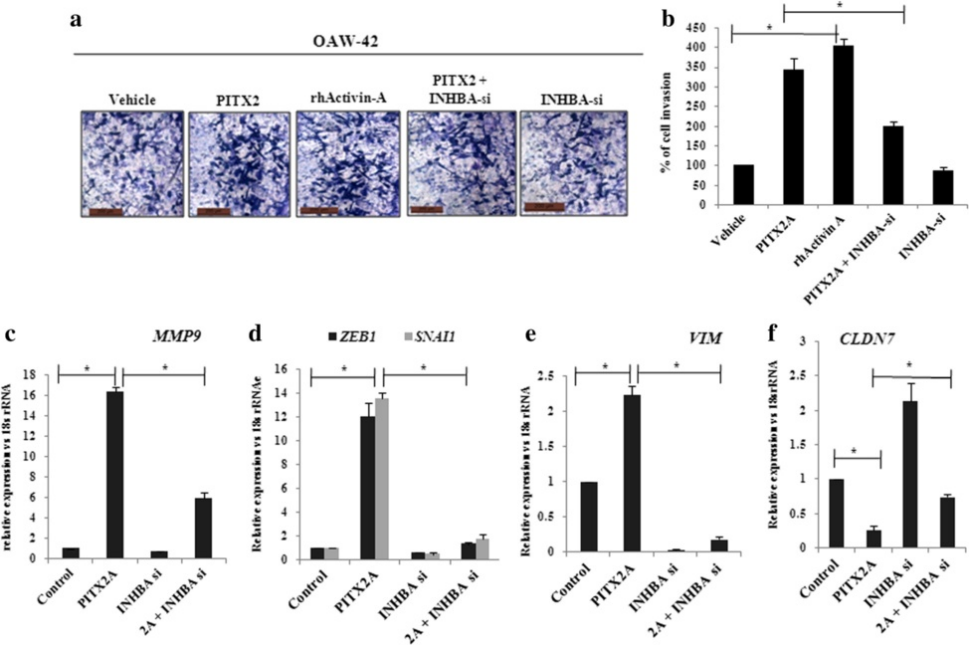
PITX2 acts through Activin-A to affect the cellular invasion and EMT. **a** Matrigel transwell assay was performed in OAW-42 cells which were treated either with rhActivin-A or transfected with *INHBA*-siRNA in presence or absence of *PITX2A*-overexpression and the percentage of cell invasion was calculated. Scale bar 100 μm. **b** Cells at three independent fields for each well were counted and plotted with error bar. **c-f** RNA was isolated from cells tarnsfected with *INHBA*-siRNA in presence or absence of *PITX2A-* overexpression and the expression of *MMP9, ZEB1*, *SNAI1*, *VIM, CLDN7* was quantified by Q- PCR with specific primers. Relative gene expression is indicated as ‘fold’ change in the Y-axis (mean ± SEM). The statistical analysis is done as described previously.*represents *p* < 0.05

We next attempted to investigate the possible contribution of activin-A in PITX2-induced invasion and EMT in ovarian cancer cells. rhActivin-A treatment resulted in ~4 fold increase in invasion of OAW-42 cells compared to controls as shown by matrigel invasion assay (Fig. 7a-b). In contrast, PITX2A-enhanced cell invasiveness was reduced by ~ 50 % on transfection with *INHBA*-siRNA (Fig. 7a-b). PITX2A increased mRNA levels of *MMP9* (Fig. 7c), *SNAI1* and *ZEB1* (Fig. 7d) that were significantly reduced by transfection with activin-A siRNA. Similarly, the mRNA levels of the mesenchymal marker *VIM* was up-regulated by ~ 2.5 folds on PITX2A over-expression, whereas it decreased drastically upon activin-A knockdown in presence of PITX2A over-expression (Fig. 7e). Knockdown of *INHBA* rescued the PITX2A-mediated suppression of the epithelial marker *CLDN7* (Fig. 7f).

## Discussion

High mortality rates of ovarian cancer patients are not only due to the rapid resistance acquired by the tumor cells to conventional therapies but also due to their overtly aggressive and invasive behaviour [29]. Metastatic cells undergo various genetic and epigenetic changes that increase motility and invasive behavior [29]. Evidence supports a strong positive role of both TGF-β ligands and their receptors in promoting carcinogenesis through invasive transformation. In tumor cells, increased secretion of the TGF-β ligands enhance metastasis and promotes tumorigenesis [30, 31]. The three mammalian isoforms, TGF-β1/β2/β3, are encoded by different genes which function throughtrans-membrane receptors with intrinsic cytoplasmic serine-threonine kinase domains [32]. Among those, TGF-β1 is most frequently up-regulated in tumor cells [33] and is the focus of most studies, including ours.

TGF-β/SMAD signalling pathway has been earlier demonstrated to be operational in ovarian cancer cells [34] and our present report demonstrated its activation in human ovarian tumors (Fig. 1). We showed up-regulation of a homeodomain transcription factor, PITX2, in similar tissue sections [28], indicating its possible involvement in ovarian cancer. Recently the association of PITX2 with cancers of thyroid, prostate, colon and breast [26, 27, 35] has been highlighted. In addition, inactivation of PITX2 leads to apoptosis of pituitary gonadotrophs [36] suggesting an active association between PITX2 and cancer, although no such report is available in the context of ovarian cancer. Considering the importance of the regulation of TGF-β pathway by homeodomain proteins including HOXB9 in promoting tumorigenesis [37], we investigated the possible association of PITX2 and TGF-β signalling in ovarian cancer. Our experiments demonstrated that PITX2 isoforms differentially enhanced the expression of *TGFB1/2/3* genes. In addition, PITX2-activated TGF-β signalling pathway (Fig. 1) was evidenced by p-SMAD2 induction as well as its nuclear localization by PITX2-CM in ovarian cancer cells.

We further investigated the role of PITX2-activated TGF-β pathway in EMT and invasion, the key steps in ovarian cancer progression [38]. We showed a significant regulatory role of PITX2 in inducing EMT through expression of EMT markers (Fig. 2) and phenotypic changes in PITX2-overexpressed cells. Earlier studies demonstrated the regulatory function of HoxB7 and HoxA10, the homeodomain transcription factors, in controlling EMT [21, 39]. Here we show an important role of PITX2 in promoting EMT. In addition, Snail and Slug are important effectors for invasiveness. They act through transcriptional repression of E-cadherin, thereby [40] facilitating metastasis [41] through basement membrane degradation, associated with lower overall survival in ovarian cancer [42]. Moreover, ectopic expression of Snail or Slug results in enhanced invasiveness and tumorigenicity in the SKOV-3 cells [43]. We showed up-regulation of Snail/Slug and downregulation of E-cadherin upon over-expression of PITX2 isoforms (Fig. 2). The activity of MMPs has been implicated in tumor growth and metastasis through ECM remodelling [44] and can be considered as an independent prognostic marker [45]. We found up-regulation of *MMP9* by PITX2 in ovarian cancer cells, suggesting PITX2 as a key regulator of relevant genes that control invasion/metastasis of ovarian cancer cells.

Our data suggests that either recombinant TGF-β1 or PITX2 over-expression can induce motility/invasion in ovarian cancer cells (Fig. 4). When PITX2-transfected cells were treated with rhTGF-β1, a synergistic activity on cellular invasion was observed. Quite surprisingly, PITX2-transfected cell lysates showed higher level of p-SMAD2 compared to rhTGF-β1-treated ones (Fig. 1c-d). We postulated involvement of some other factors up-regulated by PITX2-overexpression that could increase p-SMAD2. Indeed, we found activin-A as one such factor that is enhanced by PITX2. In addition, PITX2 binds to the bicoid-like elements present in the promoter of *INHBA* gene and trans-activates it, enhancing the formation of activin-A (Fig. 3). In ovary, activin is predominantly expressed in the granulosa cell layer of follicles and plays important roles in several physiological processes, including folliculogenesis, steroid hormone production, and oocyte maturation by acting either as a paracrine or autocrine factor [46]. Through the activation of activin signaling mediated by ActRIB-Smad2 system, activin-A facilitates the expression of FSH receptor and aromatase activity which is essential for ovarian granulosa cell function, differentiation and folliculogenesis [47, 48]. Both experimental and clinical studies suggest the elevated level of activin-A in the serum of granulosa cell tumor patients [49]. Our finding could add another dimention to the function and regulation of activin-A, particularly, in epithelial ovarian cancer.

We showed earlier that PITX2 interacts with and regulates, *FGF16*, a prime inducer of invasion of ovarian cancer cells [28]. We also showed significant involvement of PITX2 in regulating the Wnt signalling pathway that induces proliferation of ovarian cancer cells [50], creating an auto-regulatory feedback loop. The present study is the first comprehensive investigation on the regulatory role of PITX2 on invasive behavior in ovarian carcinoma cells. TGF-β pathway is considered to be a prime regulator in inducing invasion of ovarian cancer cells. Here, we identified a novel regulatory role of PITX2 through expression of both *TGFB* and *INHBA* genes. The involvement of activin-A, the homodimer form of *INHBA*, in inducing invasion leading to epithelial ovarian cancer progression (apart from GCT) has been demonstrated for the first time in this study. Thus, it is imperative to say that PITX2 appears to act as an important regulatory protein that controls initiation and progression of epithlial ovarian cancer. Further study may explore the possible involvement of PITX2 in GCT. TGF-β has been shown to synergize with oncogenic pathways. Evidence suggests that Wnts can also cooperate with other signaling pathways during tumorigenesis. PITX2 lies upstream of these two pathways, thus playing a significant role in tumorigenesis and metastasis.

## Conclusion

The major findings of our study are: a) PITX2 up-regulates the expression of ligand genes of TGF-β superfamily, *TGFB* and *INHBA* followed by induction of SMAD2/3-dependent TGF-β signalling pathway; b) through the activation of TGF-β pathway, PITX2 regulates the expression of genes that enhances invasion and EMT of ovarian cancer cells; c) This is the first report to show the direct involvement of activin-A, the homodimer form of INHBA, in promoting invasion and EMT of both non-cancerous and cancerous cells. (d) Overall this present report is first of its kind to show the direct involvement of a homeodomain transcription factor, PITX2, in the progression of ovarian cancer.

## Methods

### Cell culture, treatment of growth factor and inhibitors

Human ovarian adenocarcinoma cells, SKOV-3 (ATCC, USA) and OAW-42 (Sigma-Aldrich; USA) were maintained in McCoy’s 5A (Sigma) and DMEM (Invitrogen) respectively; both supplemented with 10 % fetal bovine serum (FBS, Invitrogen, USA), 100 U/ml penicillin and 100 μg/ml streptomycin both Invitrogen; [51]. Human **i**mmortalized **o**varian **s**urface **e**pithelial cells, IOSE (a kind gift from Drs. N. Aueresperg and Clara Salamanca, Vancouver, Canada) was maintained in Medium199 (Invitrogen) and MCDB105 (Sigma-Aldrich; USA) in 1:1 ratio supplemented with 10 % FBS, 100 U/ml penicillin and 100 μg/ml streptomycin. Here, the low-passage cultures of human ovarian surface epithelium cells (isolated by scraping from human ovarian surface tissue) were immortalized by transfecting with SV40 large-T antigen viral particles [52].

Human recombinant TGF-β1 (rhTGF-β1; Calbiochem, Germany) was used at 10 ng/ml for 30 min and 8 h for Western-blot and Q-PCR assay respectively. TGF-β receptorI kinase inhibitor (TGFRI; 20 ng/ml) was procured from Calbiochem (cat. no. 616451). Recombinant human Activin-A (rhActivin-A; ACRO Biosystems) was used at 100 ng/ml for 30 min and 24 h for Western-blot and Q-PCR assay respectively. Activin-A neutralizing antibody (Novus Biologicals) was used at a concentration of 2 μg/ml. Prior to each treatment, the cells were serum-starved for 16 h and the control cells were treated with vehicles (0.1 % BSA in 1X PBS or DMSO).

### Expression and reporter constructs

The 1.7 kb upstream promoter region of *INHBA* gene was amplified by PCR using human genomic DNA as template and then cloned into pGL3 basic vector (Promega) at MluI/HindIII site. The primer sequences used to clone the promoters are mentioned in Table 1, where the restriction enzyme sites are underlined. All constructs were sequenced by ABI Prism Automated DNA Sequencer (Perkin Elmer, USA). Sequence alignment and data analysis were performed through BLAST search (NCBI Gen Bank). The TGF-β inducible p3TP-Lux luciferase reporter vector was procured from Addgene, USA. pRL-CMV (Promega, USA) vector with Renilla-luciferase gene was used to normalise the luciferase activity. Expression plasmids of three isoforms of *PITX2* (*PITX2A/B*/*C*) were described earlier [28]. To evaluate the expression profile of the *TGFB* genes as well as the genes associated with invasion and EMT, we performed over-expression of three isoforms of PITX2 (*PITX2A/B*/*C*). As a similar trend was observed in the change in gene expression by these isoforms, we used *PITX2A* isoform as representative in the subsequent physiological experiments like activation of TGF-β pathway, matrigel invasion, wound healing and cell proliferation assay etc.

**Table 1:** The sequence of the oligonucleotide primers used to amplify specific regions of the promoter

Gene Name & Acc no	Region amplified	Forward primer (5′- 3′)	Reverse primer (5′- 3′)	Amplicon size (kb)	Tm (°C)
*INHBA*; NC_000007.14	41702063 - 41700378	GGACGCGTGAACGCTTTAACAGATGGAC	GGAAGCTTGCAAAAGTTGTTGTGATTGC	1.7	54

### Transient transfection and luciferase assay

For reporter assay, 5 × 10^4^ cells were seeded on 12-well culture plates. After 24 h, p3TP-Lux vector (0.4 μg) was transiently transfected alone or along with *PITX2* expression vectors (0.4 μg) with Lipofectamine 2000 (Invitrogen). After 4 h of transfection, the medium was replaced with fresh incomplete one supplemented with either TGFRI or DMSO for next 16 h. Each transfection was normalized with pRL-CMV vector (0.04 μg). In the following day, cells were harvested and firefly/renilla luciferase activity was determined [50]. The transfection of pGL3-reporter vectors and the subsequent assay was performed following the same protocol. Each transfection was performed in triplicate and the experiments were repeated thrice.

To over-express *PITX2* isoforms, 1 μg of expression constructs were transfected per 10^5^ cells/well in 6-well plate using Lipofectamine 2000 (Invitrogen). After 24 h and 48 h of transfection, the cells were harvested to isolate RNA and protein respectively. For the treatment of rhTGF-β1/TGFRI, the medium was replaced after 4 h of PITX2 transfection with fresh one supplemented with these factors. The cells were harvested after 16 h for RNA/protein isolation. To collect conditioned medium (CM), *PITX2A* was transiently transfected as mentioned earlier. After 6 h, the medium was replaced with fresh serum-free one, which was collected after 24 h of transfection and added directly or in combination with rhTGF-β1/TGFR1 to the freshly plated cells. The treated cells were harvested after 2 h for protein isolation. The control cells were transfected with empty vector (pcDNA3.1 Myc-His) in each case.

### siRNA and transfection

The siRNAs against *SNAI1, MMP9* and *INHBA* (all from Santa Cruz Biotechnology, USA) were used at 20 nM/well using 2 μl Lipofectamine RNAiMAX (Invitrogen) in the cells seeded in 6-well plates. The RNA interference against *PITX2* was carried out by the ON-TARGET plus SMART pool siRNA at 20 nM/well using 2 μl of Dharmafect-1 transfection reagent (Dharmacon) in cells seeded in 6-well culture plates. After 48 h of transfection, the cells were harvested for RNA/protein isolation. When required, rhTGF-β1 was added after 24 h of respective siRNA transfection into the cells.

### Quantitative Real-time RT-PCR (Q-PCR)

Total RNA was isolated from ovarian cell lines using TRI-reagent (Sigma) following the standard protocol [53]. First-strand cDNA synthesis followed by Q-PCR assay was performed as described [28]. The comparative C_T_ method (ΔΔC_T_) was used to measure relative gene expression where the fold enrichment was calculated as: 2 − ^[*Δ*CT (sample) − *Δ*CT (calibrator)]^. Here, ΔC_T_ is the C_T_ of target gene subtracted from the C_T_ of the housekeeping gene [50]. The primer sequences are mentioned in Table 2.

**Table 2:** The sequence, respective amplicon size and Tm of the oligonucleotide primers used in Q-PCR

Gene Name	Forward primer (5′- 3′)	Reverse primer (5′- 3′)	Amplicon size (bp)	Tm (°C)
18S rRNA	GATTCCGTGGGTGGTGGTGC	AAGAAGTTGGGGGACGCCGA	134	60
*POLR2A*	TGGACCCACCGGCATGTTCT	GCCCCTGGGGTCATTCCACT	141	60
*CLDN4*	GGCTGCTTTGCTGCAACT	CAGAGCGGGCAGCAGAATAC	110	60
*CLDN7*	GTGGCAGATGAGCTCCTATGC	CATCCACAGCCCCTTGTACA	80	60
*DSM*	GCAGCAAAGGGCGGAGAT	TGTTAATGTGCTGCTCCACTGA	80	60
*VIM*	ACACCCTGCAATCTTTCAGACA	GATTCCACTTTGCGTTCAAGGT	80	60
*FN1*	CCTTCATGGCAGCGGTTT	AGCGTCCTAAAGACTCCATGATCT	94	60
*PITX2*	CGCGAAGAAATCGCTGTGT	CGACGATTCTTGAACCAAACC	78	58
*TGFB1*	GTGACAGCAGGGATAACACACTG	CATGAATGGTGGCCAGGTC	80	60
*TGFB2*	GCTGAGCGCTTTTCTGATCCT	CGAGTGTGCTGCAGGTAGACA	80	60
*TFGB3*	CACCACAACCCTCATCTAATCCT	CCTGGCCCGGGTTGTC	100	60
*SNAI1*	TCGGAAGCCTAACTACAGCGA	AGATGAGCATTGGCAGCGAG	140	60
*SNAI2*	ATGAGGAATCTGGCTGCTGT	CAGGAGAAAATGCCTTTGGA	119	60
*CDH1*	GTCACTGACACCAACGATAATCT	TTTCAGTGTGGTGATTACGACGTA	100	60
*MMP9*	ACCTCGAACTTTGACAGCGAC	GAGGAATGATCTAAGCCCAGC	113	60
*INHBA*	GTGAGTGCCCGAGCCATATAG	CATGCGGTAGTGGTTGATGACT	80	60
*CDH2*	CCATCAAGCCTGTGGGAATC	GCAGATCGGACCGGATACTG	76	60
*ZEB1*	CAATGATCAGCCTCAATCTGCA	CCATTGGTGGTTGATCCCA	117	60
*ZEB2*	AAGCCCCATCAACCCATACAAG	AAATTCCTGAGGAAGGCCCA	124	60

### Chromatin-immunoprecipitation (ChIP)

ChIP with SKOV-3 cells was performed following the methods described earlier [50]. For ChIP-PCR, the immunoprecipitated (IP) and input DNA were used at equal quantity following the conditions: 95 °C for 30 s, annealing at specific temp for 30 s and extension at 72 °C for 30 s, for 30 cycles. The information of the primers is shown in Table 3.

**Table 3:** The sequence, respective amplicon size and Tm of the oligonucleotide primers used in ChIP-PCR

Gene Name & Acc no	Region amplified	Forward primer (5′- 3′)	Reverse primer (5′- 3′)	Amplicon size (kb)	Tm (°C)
*INHBA*; NC_000007.14	41702097- 41701831	GGCTTATGTGTGGGAAAGAA	ACCAGTGCATTCATAGACAG	265	55.5

### Western blot analysis

Cell lysis and protein extraction was performed as described previously [51] and subjected to immunoblotting with antibodies specific for the proteins including, PITX2 (Chemicon, 1:1000), α-SMA (Sigma; 1:1000 dilution), claudin-7, MMP9 (both Santa Cruz; 1:1000 dilution), GAPDH (1:3000), E-cadherin, SNAIL, vimentin, p-SMAD2 and SMAD2 (all 1:2000 dilution; all from Cell signalling technology, USA).

### Confocal microscopy

Immunofluorescence staining with anti-p SMAD2 (1:100) E-cadherin (1:100) and PITX2 (1:100) antibodies followed by Alexa-fluor 488-conjugated secondary antibody was performed as described previously [28]. For phalloidin staining 10^5^ cells/well were plated in 6-well plate. After 24 h of *PITX2A* transfection or rhActivin-A treatment, actin filament bundle formation was observed by phalloidin staining as mentioned previously [54] followed by imaging with confocal microscopy.

### Wound healing assay

Serum-starved cells at 70 % confluency were transfected with *PITX2A*-construct. After 4 h, medium was replaced by fresh and incomplete one supplemented with rhTGF-β1/TGFRI. In additional experiment, treatment was also given in other set of serum-starved cells to check the effect of only TGF-β1/TGFRI on migration. Scratching was carried out with a 200 μl pipette tip prior to the treatment and mentioned as t = 0 h at the figure. Cells were washed several times with PBS to remove the detached ones and supplied with new growth medium. Photographs of the scratches were taken at 0 and 24 h using an inverted microscope (Leica) equipped with a Scion digital camera and in-built software (Leica application suite v3.0).

### *In vitro* invasion assay

Transwell membranes coated with Matrigel (BD Biosciences, USA) were used to assay *in vitro* invasion as mentioned previously [53]. In brief, 2.5 × 10^5^ cells were seeded in the upper chamber in serum-free medium and FBS or rhTGF-β1/TGFRI was added in the lower chamber. To check the effect of *PITX2, MMP9* and *SNAIL* on TGF-β-mediated invasion, cells were transiently transfected with respective construct or siRNAs on previous day, allowed to recover overnight and then serum-starved for additional 16 h. The cells were then trypsinized, counted and equal number of transfected cells were added in the upper chamber and allowed to invade in presence of TGF-β or TGFRI.To understand the role of activin-A in PITX2-mediated cell invasion, the cells were transiently transfected with *PITX2* expression construct or *INHBA*-siRNAs or both on previous day, allowed to recover overnight and then serum-starved for additional 16 h. The cells were then trypsinized, counted and equal number of transfected cells were added in the upper chamber and allowed to invade in presence or absence of rhActivin-A. After incubating for 22 h at 37 °C in 5 % CO_2_, the invaded cells were fixed, stained and counted under microscope. Three independent experiments were performed followed by statistical analysis.

### Immunohistochemistry (IHC) with immunofluorescence (IF)-based detection

Tissue sample blocks used for IHC were archival materials provided by the Department of Pathology, Institute of Post Graduate Medical Education and Research and SSKM Hospital, Kolkata, India. Isolated tissues were fixed, processed, and sectioned as mentioned earlier [55]. The sections were then blocked in 5 % BSA in 1XTBS-T for 30 min and incubated for 2 h with the anti-pSMAD2 (1:100) and antibody diluted in 1XTBS containing 0.1 % BSA. The slides were then washed and incubated for 1 h with the secondary antibody (Alexa Fluor-488; 1:500) followed by staining with DAPI. The IF-staining was performed in all collected samples and the representative images have been shown.

### Statistical analysis

All data were expressed as mean ± SEM and the ± SEM are represented by error bars. The statistical significance was calculated by two-tailed Student’s *t*-test. *p* < 0.05 was considered as significant. The experiments were done at least 3 times in duplicate unless otherwise stated.

## Additional file

Additional file 1: Figure S1. The expression of PITX2 is up-regulated in human ovarian cancer. (A) The level of PITX2 was observed by IHC in human ovarian tissue-sections with specific antibody followed by Alexa Fluor-488 (green) of low malignant potential (i; *n* = 20) and high malignant potential metastatic adenocarcinoma patients (ii; *n* = 20). The DAPI-stained nuclei and the merged images were also shown. Scale bar, 10 μm. (TIFF 28495 kb)

## Abbreviation

DAPI: 4′, 6-Diamidino-2-phenylindole
TGF: Transforming growth factor
MMP: Matrix metalloprotease
SMAD: Mothers against decapentaplegic, Drosophila
EMT: Epithelial-mesenchymal transition
GAPDH: Glyceraldehyde-3 phosphate dehydrogenase
ECM: Extracellular matrix
SMA: Smooth muscle actin
CLDN: Claudin
CDH1: E-cadherin
CDH2: N-cadherin
*SNAI1*: SNAIL
*SNAI2*: SLUG
*INHBA*: activin-A
IOSE: Immortalized ovarian surface epithelium
