# Supplementary figures and images for: Invasion of ovarian cancer cells is induced byPITX2-mediated activation of TGF-β and Activin-A
Source: Mol Cancer. 2015 Aug 23;14:162. doi: 10.1186/s12943-015-0433-y (PMC4546816; doi:10.1186/s12943-015-0433-y)

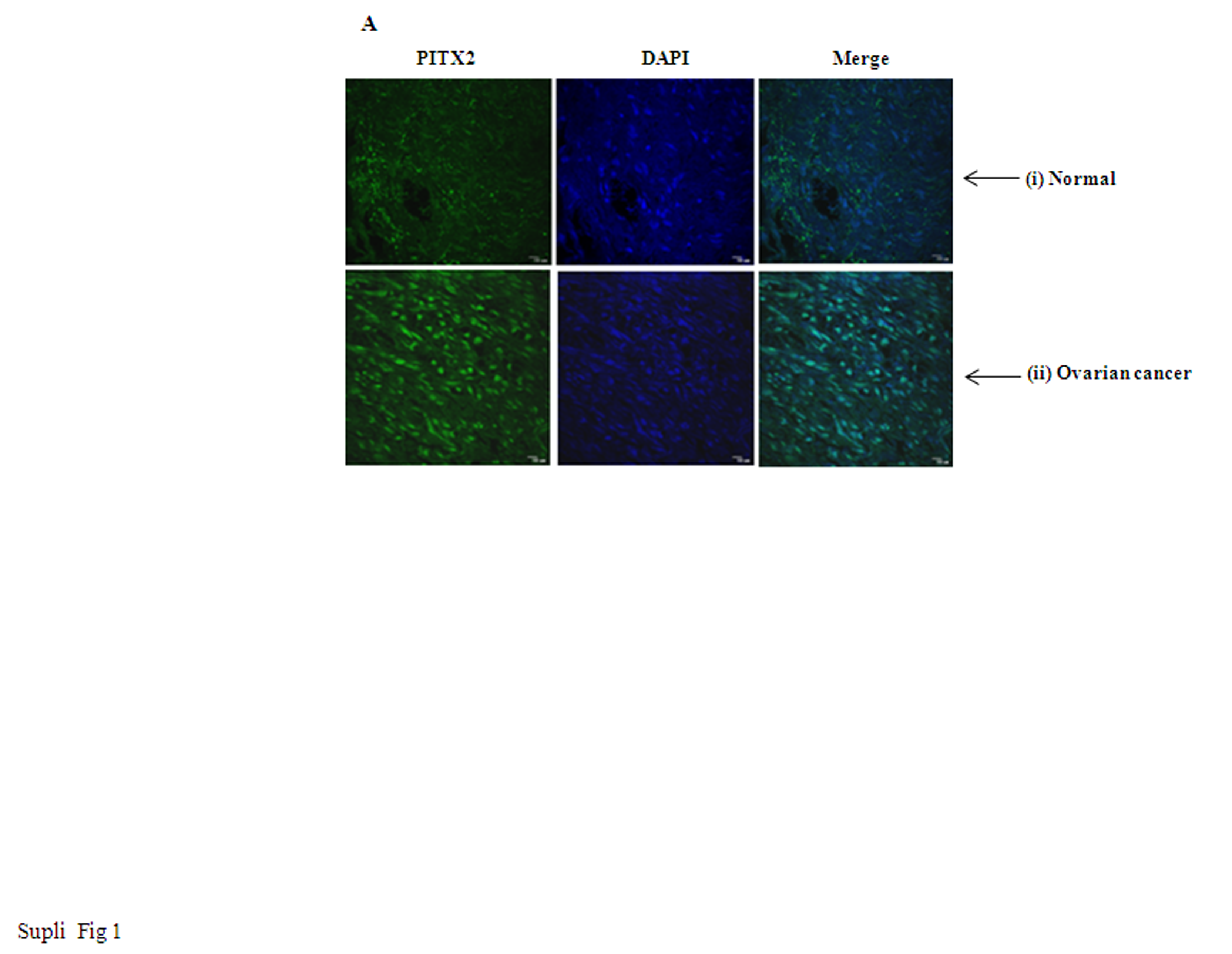

Supplement: Additional file 1: Figure S1. — The expression of PITX2 is up-regulated in human ovarian cancer. (A) The level of PITX2 was observed by IHC in human ovarian tissue-sections with specific antibody followed by Alexa Fluor-488 (green) of low malignant potential (i; n = 20) and high malignant potential metastatic adenocarcinoma patients (ii; n = 20). The DAPI-stained nuclei and the merged images were also shown. Scale bar, 10 μm. (TIFF 28495 kb) [file 12943_2015_433_MOESM1_ESM.tif]
